# Stunting in the Context of Plenty: Unprecedented Magnitudes Among Children of Peasant's Households in Bukombe, Tanzania

**DOI:** 10.3389/fnut.2019.00168

**Published:** 2019-11-07

**Authors:** Lucas L. Shilugu, Bruno F. Sunguya

**Affiliations:** School of Public Health and Social Sciences, Muhimbili University of Health and Allied Sciences, Dar es Salaam, Tanzania

**Keywords:** stunting, feeding practices, peasant, dietary diversity, food security

## Abstract

**Background:** It is perceived that children living in peasants' households are protected from undernutrition owing to a relative better food availability. However, evidence suggests an increased vulnerability that is not conforming to such norm and varies from one region to another. To address this research gap, we examined the magnitude and factors associated with stunting among under-5 children from peasant's households and compared them with children of other households in a rural district in Tanzania.

**Methods:** This cross-sectional study was conducted in Bukombe district, Tanzania, among the randomly selected 358 under-5 child-caregiver pairs. We collected data through face-to-face interviews and took anthropometric measurements, which were converted to height for age Z-score. Data were analyzed using both descriptive and logistic regression methods to compare the nutrition status of children in two contexts and determine other factors associated with stunting among children in Bukombe district.

**Results:** Under-5 children in Bukombe district succumbed to a higher magnitude of stunting (52.8%) compared to the national average. In comparison to the children from the other households, those residing in peasant households succumbed to even higher burden of stunting (46 vs. 56%). Poor feeding practices were common in these communities and more pronounced among peasant communities. About 71% of children in peasants' households had lower dietary diversity compared to 55% of other households (*p* = 0.003). Other factors associated with stunting included older age (AOR = 2.74, *p* = 0.003), severe food insecurity (AOR = 3.34, *p* = 0.002), and birth weight (AOR = 0.31, *p* = 0.02).

**Conclusion:** Children of peasants' households in Bukombe district are at a higher risk of stunting compared to households with other occupations despite their engagement in farming. In addressing this persistent challenge in rural Tanzania and areas with similar context, efforts should be streamlined to address poor feeding practices, food insecurity, and the interventions tailored for maternal nutrition to ameliorate low birth weight.

## Introduction

Undernutrition among children under-5 is a major public health concern in developing countries (1–3). It results in far-reaching consequences later in life (4) as it affects growth and cognitive development. Undernutrition remains a factor to more than one third child mortality globally (5, 6) and consequently the most important risk factor for the burden of disease in developing countries (7). The first 1,000 days are crucial in development, and efforts to address risk factors during this period have the potential to ameliorate undernutrition and the long-term consequences resulting from such conditions (5, 8–10). The burden of undernutrition is higher in low- and middle-income countries, and Tanzania is no exception. Tanzania is among 10 countries with the highest burden of stunting. Efforts to address this burden have resulted into a decline from about 50% in 1990s to 34% in 2015 (1, 3). The remaining burden is still high and risking the nation's development agenda.

Food insecurity is one of the underlying causes of undernutrition. However, contrary to the global trend, the magnitude of stunting in Tanzania remains persistently higher in high food-producing regions (2, 11). Such regions known as “food basket regions” of Tanzania include Ruvuma, Iringa, Njombe, and Rukwa. These and others with relatively higher food productivity including Geita and Kagera regions succumb to unprecedented prevalence of stunting above 40% (1, 2). Evidence generated from some of these regions suggested that women involvement in farming increases the risk of undernutrition (3). These primary caregivers use most of their daytime in farms, an occupation that deprives child caring including breastfeeding. Children in these areas are weaned much earlier than recommended to allow their caregivers return to their farming activities (3, 12).

Notwithstanding their engagement in farming and therefore food productivity, peasants' households may face food insecurity owing to including seasonal food insecurity, post-harvest losses, poverty, poor nutritional knowledge, and extended cycle of demographical disadvantages. Most of these peasants grow only one or two types of food products, subjecting them into low dietary diversity and therefore insecurity. To a larger extent, such challenges may contribute to a higher burden of stunting. To ameliorate it, tailored nutrition sensitive and specific interventions are needed to address the risk factors. So far, determinants of stunting among children in peasant families have not been studied widely, and there is a dearth of information on how stunting is associated with the occupation in which the household is engaged. We therefore sought to examine the magnitude of stunting among under-5 children in peasant households and compared to that of children from families engaging in other economic activities. We also assessed the other local determinants of stunting among children in rural Bukombe district.

## Methods

### Study Design and Setting

This cross-sectional study examined the magnitude of stunting, feeding practices, and other factors associated with stunting among children under-5 in peasant families in rural Bukombe district, Tanzania. We also assessed magnitudes of other nutrition status including underweight and wasting stratified by occupation status of the household. Bukombe district is one of six districts in Geita region in the Northern Tanzania. Stunting is prevalent to 41% of children under-5 (2). The major economic activity of residents in this district is small-scale farming. Others engage in petty trade, small-scale mining, formal/skilled employment, and self-employment through different unskilled manual works (13). Data were collected in July and August 2018, coinciding a post-harvest season in the area.

### Participants

We recruited a total of 358 under-5 children-caregiver pairs. We randomly sampled four out of the 17 wards. We selected four villages from each ward and sampled 22 or 23 households per village to give 358 study households through a systematic random sampling. In case a selected household had more than one child under the target group, a simple random sample using paper numbers was used to select one child. In case a sampled household had no child under the study target age group, the nearest house was used to replace the household.

### Measurements

The outcome variable was stunting status defined as children below minus two standard deviations (−2SD) of the height for age Z-score (HAZ) in the reference population. Other undernutrition measures were wasting and underweight. Children below −2SD of the standard population's weight for height Z-score (WHZ) were regarded wasted while those below −2SD of the standard population's weight for age Z-score (WAZ) were considered underweight (14). We used the 2011 WHO Anthro software version 3.2.2 to calculate HAZ, WHZ, and WAZ.

Independent variables included child feeding practices measured through feeding frequency and dietary diversity. Assessment of the feeding frequency was through a question to the caregiver on a number of times they fed their children in the previous 24 h (12). Responses of below four times per day were categorized as low feeding frequency. To assess dietary diversity, caregivers were asked to identify the food type the children were fed in the previous 24 h. A list of common food in Bukombe was prepared in line with the nationally representative survey questionnaire. A list of eight food groups provided by Food and Nutrition Technical Assistance (FANTA) tool (15) was used to form the child dietary diversity score (DDS). Minimum dietary diversity was referenced from the nationally representative survey 2015–2016, that is, feeding from at least four out of the following eight food groups: grains, roots, and tubers; legumes and nuts; dairy products (milk, yogurt, and cheese); flesh foods (meat, fish, poultry, and liver/organ meat); eggs; Vitamin A-rich fruits and vegetables; other fruits and vegetables, and food cooked in oils/fats. Consumption of food from at least four food groups means that the child has a high likelihood of consuming at least one animal source of food and at least one fruit or vegetable in addition to a staple food (grains, roots, or tubers) (2).

Household food insecurity was assessed using Household Food Insecurity Access Scale (HFIAS) in the past 1 month basing on the nine-item questionnaire provided by FANTA (16, 17). In this study, the HFIAS had a Cronbach's alpha of 0.89 and an item-to-rest correlation ranging from 0.87 to 0.9. The scores were grouped into food secure, mildly insecure, moderately insecure, and severely food insecure (16, 17) like in another study conducted in Tanzania (12).

We assessed illness episodes by asking caregivers to recall whether their children had disease conditions. They included malaria, fever, skin diseases, acute respiratory infections, pneumonia, vomiting, or diarrhea in the past 1 month. We measured birth intervals for children who had siblings at the time of the study by asking the caregivers to recall the time when the sibling was born. Responses were categorized into below 24 months or above 24 months (18).

To assess antenatal visit, caretakers were asked to recall the number of antenatal clinics the mother had during pregnancy of the child. The responses were categorized into three or less visits as low number of visits, and four and above as the required number of visits as recommended by the WHO and the Tanzania Ministry of Health and Community Development, Gender, Elderly and Children (MOHCDGEC) guidelines as also applied in national surveys (2). To assess post-natal health checks for newborns, we based on the TDHS-MIS 2015/16 questionnaire as having received any health facility post-natal health checks. We measured child immunization status (19) defined as full immunized or not completed vaccination as per the recommended schedule by the MOHCDGEC available and applied in the TDHS-MIS 2015/16 (2). Completion of Penta-3 vaccine was the indicator for completion of vaccines (2).

To assess birth weight, we obtained information in the child's Reproductive and Child Health (RCH) card number 4 used to monitor child growth, immunization, and clinic attendance. As recommended by the WHO categories for birth weight were below 2.5 kg as low birth weight, between 2.5 and 3.5 kg as normal, or above 3.5 kg as high birth weight as also applied in the national survey (2). We defined place of delivery as applied in the national survey (2), categorized that as health facility delivery or home/way delivery.

We categorized caregiver education level according to Tanzania education systems as also applied in another study (12) and categorized into no formal education, having a primary level education, or having above primary level. We measured family economic activities by asking caretakers to self-report the main occupation of the household. Responses were based on the main economic activities common in the area that included farming, petty trade, food seller, bodaboda (a public transport system using motorcycle), small mining scale, formal employment (in the government or other annual contracted jobs in registered organizations), informal employment (unskilled labor) or unregistered example day workers. In analysis, five categories of occupations (farming, employees, businessman/woman, small mining, and unskilled manual labor) were maintained.

The weighted wealth index was calculated using household's ownership of household items; housing characteristics such as source of drinking water, toilet facilities, and flooring materials; and food availability. These dichotomized variables were adopted from the household's questionnaire of the TDHS-MIS 2015/16 (2). The dichotomized variables were reduced using principal component analysis (PCA) from 52 initial variables to 19 that loaded as the first output component with 45% of the variation that may closely measure economic status. Factor loadings were summed and categorized into five equal wealth quintiles as poorest, poor, middle, rich, and wealthiest.

### Data Collection

We collected anthropometric data using SEGA digital scale for measuring child weight as recommended by the WHO (20) and like in other studies (2, 3). For the weighing of very young children who could not stand alone on the scale, the mother or caretaker was weighed first, then the mother or caretaker was weighed again while holding the child after taping the mother-baby button (tarred weighing); the child weight showed on the screen and recorded in kilograms. Height was measured in centimeters using a wooden length measuring board. Younger children below 24 months and who could not stand were measured lying down beside the board (recumbent length), while standing height was measured for older children (2, 3).

A pretested and translated questionnaire from English to Swahili language was used to collect data from caregivers. We recruited research assistants from community health workers with data collection experience. They had primary level education or above and were working in the same district on health-related projects. We conducted training for 2 days to familiarize them with the aims of the study, the tools and interpretation of questions, ethical consideration, and use them to conduct the pretesting of the tool. Of the 2 days, the first day training was conducted in the class, while the second day was field practical training.

### Data Analysis

Data was analyzed using both descriptive and regression analyses. For descriptive analyses, we examined the characteristics of the study population including the demographic characteristics, feeding practices, burden of illnesses, and the nutrition status. We used chi-square test to compare such characteristics as sex, nutrition status, feeding practices, and occupation of the households. Bivariate and multiple logistics regression analyses were conducted to examine factors associated with stunting. Associations that reached *p* < 0.2 at bivariate analysis were included into the multiple logistics regression analysis.

## Results

### Sociodemographic Characteristics of Children

The mean age in the study population was 29 months (SD 15.34). Four in 10 children were from a caregiver with no formal education, and more than half of the children were from households engaging in farming as the main occupation. Above half of the children were from households that obtain food from their own farming harvests (Table 1).

**Table 1:** Descriptive characteristics of children age 6–59 months recruited for the study in Bukombe, June 2018 (*N* = 358).

**Variable**	**Total**		**Male**		**Female**		***P* Value**
	***N***	**%**	***N***	**%**	***N***	**%**	
**Age of child (months)**							
6–11	74	20.7	36	20.9	38	20.4	
12–23	64	17.8	27	15.7	37	19.9	
24+	220	61.5	109	63.4	111	59.7	0.580
**Household head's main occupation**							
Peasant	242	67.6	117	68	125	67.2	0.094
Unskilled manual labor	38	10.6	23	13.4	15	8.1	
Employee	17	4.7	7	4.1	10	5.4	
Businessman/ woman	16	4.5	10	5.8	6	3.2	
Small mining activities	45	12.6	15	8.7	30	16.1	
**Main source of food**							
Purchasing	112	31.3	52	30.2	60	32.3	0.367
Own crop harvest	241	67.3	116	67.4	125	67.2	
Supplies from relatives	5	1.4	4	2.3	1	0.5	

### Feeding Practices and Nutrition Status Among Children

About three in every four under-5 children on complementary feeding had low feeding frequency, while 65.4% had low DDS. More children with low DDS were from peasant families (70.6%) compared to (54.5%) children from families in other occupations (*p* = 0.003) (Table 2). More than half of the children in this study (56.2%) were stunted. Magnitude of stunting was higher (56.2%) among children from peasant families compared to (45.7%) children from families engaged with other occupations (*p* = 0.062) (Table 2).

**Table 2:** Household occupation distribution of feeding practices and nutrition status among children age 6–59 months recruited for the study in Bukombe, June 2018.

**Child feeding practices**	**Peasants**		**Other occupations**		**Total**		***P* value**
	***N***	**%**	***N***	**%**	***N***	**%**	
**Complementary food initiation time**							
<6 months	64	26.9	39	34.8	103	29.4	
6 months	134	56.3	57	50.9	191	54.6	
≥7 months	40	16.8	16	14.3	56	16	0.311
**Child feeding frequency in 24 h**							
≤3 times	168	70.6	88	78.6	256	73.1	
≥4 times	70	29.4	24	21.4	94	26.9	0.116
**Dietary diversity score (DDS)**							
≤3 scores	168	70.6	61	54.5	229	65.4	
≥4 scores	70	29.4	51	45.5	121	34.6	0.003
**Stunting (<-2.0SD)**							
Stunted	136	56.2	53	45.7	189	52.8	
Normal	106	43.8	63	54.3	169	47.2	0.062
**Underweight (<-2.0SD)**							
Underweight	27	11.2	6	5.2	33	9.2	
Normal	215	88.8	110	94.8	325	90.8	0.067
**Wasting (<-2.0SD)**							
Wasted	8	3.3	0	0	8	2.2	
Normal	233	96.7	116	100	350	97.8	
**Household food security**							
Food secure	115	79.9	29	20.1	144	40.2	
Mild insecurity	55	66.3	28	33.7	83	23.2	
Moderate insecurity	43	46.2	50	53.8	93	26	
Severe insecurity	29	76.3	9	23.7	38	10.6	<0.001
**Household main source of food**							
Own crop harvest	119	82.6	42	17.4	241	67.3	
Purchasing	43	36.8	74	63.2	117	32.7	<0.001

### Factors Associated With Stunting

Children from a food insecure household were more likely to be stunted [adjusted odds ratio (AOR) = 3.34, 95% CI = 1.11–10.02, *p* = 0.032]. Children aged 24–59 months were more likely to be stunted compared to younger children (AOR = 2.74, 95% CI = 1.47–5.13, *p* = 0.003). Moreover, children born with normal weight (2.5–3.5 kg) were less likely to be stunted compared to children with underweight (AOR = 0.31, 95% CI = 0.12–0.83, *p* = 0.02). Children from peasantry households had two times higher risk of stunting compared to children from households engaged with other occupations (AOR = 1.96, 95% CI = 0.96–3.91, *p* = 0.063); however, the association did not reach a statistically significant level (Table 3).

**Table 3:** Logistic regression; factors associated with stunting (HAZ <-2.0SD) among children age 6–59 months in Bukombe, June 2018.

**Variable**	**Bivariate logistic regression**			**Multiple logistic regression**		
	**OR**	**95% CI (OR)**	***P***	**AOR**	**95% CI (AOR)**	***P***
**Age (months)**						
6–11	1.0			1.0		
12–23	1.46	0.83–2.56	0.185	1.59	0.86–2.91	0.14
24–59	2.37	1.36–4.12	0.002	2.74	1.47–5.13	0.003
**Sex of child**						
Female	1.0			1.0		
Male	1.38	0.91–2.09	0.128	1.47	0.94–2.3	0.09
**Household occupation**						
Other occupations	1.0			1.0		
Peasants	1.52	0.98–2.38	0.055	1.94	0.96–3.91	0.063
**Care taker level of education**						
No education	1.0			1.0		
Primary school	0.93	0.59–1.45	0.755	1.02	0.54–1.92	0.949
Post-primary school	0.58	0.29–1.16	0.126	0.53	0.19–1.46	0.220
**Weighted wealth index**						
Poorest	1.0			1.0		
Poor	1.06	0.55–2.03	0.868	1.07	0.44–2.58	0.881
Middle	1.09	0.57–2.07	0.808	0.87	0.36–2.12	0.757
Rich	1.18	0.61–2.31	0.609	0.96	0.39–2.37	0.937
Richest	1.72	0.89–3.35	0.109	2.39	0.97–5.90	0.059
**Breast feeding initiation time**						
Within an hour	1.0			1.0		
After 1 h	1.18	0.71–1.81	0.465	1.82	0.97–3.43	0.064
**Exclusive breast feeding**						
EBF	1.0			1.0		
Non-EBF	1.02	0.67–1.55	0.930	0.94	0.53–1.66	0.826
**Breast feeding duration**						
<24 months	1.0			1.0		
≥24 months	1.13	0.65–1.95	0.666	0.91	0.47–1.75	0.775
**Feeding frequency**						
≤3 times	1.0			1.0		
≥4 times	0.74	0.47–1.23	0.269	0.59	1.29–1.19	0.142
**Dietary diversity score**						
≤3 scores	1.0			1.0		
≥4 scores	0.98	0.63–1.53	0.930	0.73	0.39–1.34	0.306
**Food insecurity**						
Food secure	1.0			1.0		
Mild insecurity	0.94	0.55–1.61	0.809	0.87	0.41–1.87	0.722
Moderate insecurity	0.72	0.43–1.21	0.212	0.82	0.40–1.69	0.600
Severe insecurity	2.14	0.99–4.63	0.055	3.34	1.11–10.02	0.032
**Child illness**						
Absent	1.0					
Present	1.58	0.7–3.53	0.269			
**Malaria**						
No episode	1.0					
Having episode	0.97	0.62–1.53	0.900			
**Diarrhea**						
No episode	1.0					
Having episode	1.29	0.21–7.86	0.781			
**Fever**						
No episode	1.0			1.0		
Having episode	1.41	0.93–2.15	0.106	1.79	0.98–3.27	0.056
**Antenatal care**						
≤3 times	1.0					
≥4 times	0.97	0.64–1.48	0.902			
**Post-natal care**						
Yes	1.0					
No	1.19	0.78–1.84	0.419			
**Vaccination**						
Completed	1.0					
Not completed	1.08	0.61–1.88	0.800			
**Low birth weight**						
≤2.5 kg	1.0			1.0		
2.5–3.5 kg	0.36	0.14–0.91	0.031	0.31	0.12–0.83	0.02
>3.5 kg	0.44	0.17–1.13	0.089	0.34	0.13–0.93	0.04
**Place of delivery**						
Health facility	1.0					
Home/way	1.04	0.68–1.6	0.845			

*OR, odds ratio (crude); AOR, adjusted odds ratio; EBF, exclusive breast fed*.

## Discussion

Magnitude of stunting was higher in this rural peasantry community compared to the national average (2). Moreover, evidence generated from this nationally representative survey suggests that children of peasants' households suffered the greater brunt of undernutrition compared to other occupations. Such high burden of undernutrition could be a result of poor feeding practices that were predominant in this context. Among children recruited for the study, 73.1% had low feeding frequency, and 65.4% had low DDSs. This is similar to 70% low feeding frequency reported among children in Geita region (2) and not very different from 53.5% reported in another rural district of southern highlands of Tanzania (3).

Low feeding frequency might be explained by caretakers' involvement in farming, taking most of their day and thus affecting child care including breastfeeding and complementary feeding (3). It may also be explained by the seasonal household's food insecurity in farming communities reported elsewhere (12). Dietary diversity was low and might be explained by the sources of food of the household. Most of such households engage in monoculture farming. Under such contexts, they may predominantly have access to a limited number of food products and mostly cereal-based food. This can be related to evidence from this study that majority of peasant households depend on food on their own crop harvests compared to households on other occupations that acquire food through purchasing. Thus, children from peasant households were likely to be fed only what is available in the harvest stocks. In the context of Bukombe, such foods are corn, millet, potatoes, and cassava, all of which provide carbohydrates and not adequate proteins among other nutrients. Children from households in other occupations were more likely to be fed what was mainly purchased, which might be of a variety of food groups.

Children in predominant peasant households were more likely to be stunted compared to their counterparts from families in other occupations. Evidence suggests that the higher likelihood of undernutrition among children in peasant households may be due to poor maternal health, poor parental care, and poor child feeding practices associated with women's long time involvement in farming activities (3, 12). Low dietary diversity was more likely among children in peasant households compared to others in this study. Lack of adequate caring time for their children may also subject them to poor health seeking behavior and access to clean water and poor sanitation, all leading to poor health and undernutrition. Stunting among children from a food insecure household was higher compared to food secure households. Household food insecurity is associated with poor child feeding practices in other contexts (12).

Low birth weight increased the likelihood of stunting. Stunting was rarer among children under normal birth weight compared to children with lower birth weight. Similarly, findings have been reported in rural districts of Tanzania (21, 22) and Kenya (23). This might reflect poor maternal nutrition and development of chronic undernutrition through transgenerational effect of *in utero* effects (2, 5, 10). Earlier studies documented that maternal nutrition is a strong factor for birth outcomes and later health status of the child (5, 21, 24). Mother's poor nutritional status before conception, short stature, and poor nutrition during pregnancy, poor feeding, and weight gain during pregnancy have higher chances of low birth weight and stunted children (5, 21, 24). In the context of this study, poor maternal nutrition and low birth weight outcomes might be explained by the household occupation. In this predominant peasant community, women are more likely to succumb to poor maternal health due to spending more time in farms with consumption of low food diversity, which is available for them. Poor health seeking behavior, inadequate nutritional care, and lack of resting time may put pregnancy at higher risk.

Older under-5 children were more likely to be stunted compared to younger ones like in previous studies (12, 18, 23). Like in other studies, stunting starts to manifest after exclusive breastfeeding period owing to introduction of low dietary diversity and frequency. Moreover, food insecurity, which is predominantly among farming communities, further subjects these children to a narrow range of potentially nutritional food substrates capable of ameliorating undernutrition and particularly stunting. In peasant households, caregivers are more likely to reduce their care time to older children in prioritizing farming activities. At age 2 years, children are expected to remain at home while mothers/caretakers go to farms, affecting parental care and child feeding practices (3).

### Potential Study Limitations and Mitigation

We used tools that required recall for as long as 1 month post-event. This might have led to loss of accuracy in information. This limitation was kept minimal by taking time with the participant and giving them time to remember events. For DDS and child feeding frequency, the recall was 24 h, illness episodes among children recall was 30 day, household food insecurity recall was 30 days as recommended by FANTA (16, 17). This study was a cross sectional in design, and evidence cannot provide causality. However, in addition to other evidence, it provides associations that can be used to provide recommendations. Lack of generalizability is another potential limitation. However, we can generalize our findings to areas with similar contexts in Tanzania and beyond.

## Conclusions and Recommendations

Magnitude of undernutrition was high in Bukombe district. Chronic form of undernutrition was prevalent in among 52.8% of all under-5 children sampled in this study. Stunting was more prevalent among children in peasant population compared to children in other occupations due to poor feeding practices. Majority of children were fed at a low feeding frequency and dietary diversity. Stunting was also significantly associated with age, household food insecurity, and low birth weight.

In addressing undernutrition among children in Bukombe district and others with similar contexts, tailored interventions should target the first 1,000 days, households with food insecurity, and children born with low birth weight. To ameliorate undernutrition among children of peasant households, interventions are needed to improve maternal nutrition emphasizing pre- and during and post-pregnancy periods, access to and attendance to antenatal and post-natal periods, and providing adequate care and time for children under-5. Knowledge in feeding practices and access to nutritional adequate food remain of paramount importance.

## Data Availability Statement

The data sets used and analyzed during the current study are available and still under analysis for subsequent publications but will be available upon request from authors.

## Ethics Statement

Ethical clearance was provided by the Muhimbili University of Health and Allied Sciences (MUHAS) Ethical Committee, issued on May 29, 2018. Permission to conduct the study at the field was requested and provided by the Bukombe district medical officer. Written informed consents were obtained from parents/guardians of children after being informed on the operation and application of the study findings. Confidentiality of the respondents was ensured at all stages of the study.

## Author's Note

LS was a postgraduate student pursuing MPH under the supervision of BS senior lecturer and the Director of Research and Publications at Muhimbili University of Health and Allied Sciences, Tanzania.

## Author Contributions

LS designed the study, conducted data collection, did data analysis and interpretation of findings, wrote and approved the manuscript. BS provided technical inputs to improve designing the study, supported data analysis, read, improved, and approved the final manuscript write-up.

### Conflict of Interest

The authors declare that the research was conducted in the absence of any commercial or financial relationships that could be construed as a potential conflict of interest.

## Abbreviations

AOR: adjusted odd ratio
CI: confidence interval
EBF: exclusive breast feeding
FANTA: Food and Nutrition Technical Assistance
HAZ: height-for-age Z-score
HFIAS: Household Food Insecurity Access Scale
HH: households
MOHCDGEC: Ministry of Health, Community Development, Gender Elderly and Children
OR: odd ratio
SD: standard deviation
SPSS: Statistical Package for Social Science
TDHS-MIS: Tanzania Demographic and Health Survey and Malaria Indicator Survey
TFNC: Tanzania Food and Nutrition Centre
WAZ: weight-for-Age Z-score
WB: World Bank
WHA: World Health Assembly
WHZ: weight-for-height Z-score.
